# Treating alcohol use disorders in primary care – a qualitative evaluation of a new innovation: the 15-method

**DOI:** 10.1080/02813432.2021.1882079

**Published:** 2021-02-15

**Authors:** Sara Wallhed Finn, Anders Hammarberg, Sven Andreasson, Maria Jirwe

**Affiliations:** a Department of Global Public Health, Karolinska Institutet, Centre for Psychiatry Research, Stockholm Health Care Services, Stockholm, Sweden; b Department of Clinical Neurosciences, Karolinska Institutet, Centre for Psychiatry Research, Stockholm Health Care Services, Stockholm, Sweden; c Department of Health Science, Red Cross University College, Stockholm, Sweden

**Keywords:** alcohol, alcohol use disorders, treatment, implementation, stepped care, qualitative method

## Abstract

**Objective:**

This study aims to explore how the characteristics of an innovation, the 15-method, a stepped care model for treatment of alcohol use disorders in primary care was perceived.

**Methods/Design/Setting/Subject:**

General practitioners and heads of primary care units (*n* = 10) that delivered the 15-method in a randomized controlled trial participated in individual interviews at two occasions in Stockholm, Sweden. Data were analyzed with theoretical thematic analysis, using Diffusion of Innovation Theory.

**Results:**

The participants described that offering the 15-method met a need among their patients. Participants were positive towards the training and the manual for the method. They mentioned a previous lack of routines to work with alcohol use disorders. The 15-method was described as easy to use. It would however be more feasible to implement in a team of different professions, rather than among general practitioners only. Priorities made by regional health care managers were described as important for the implementation, as well as financial incentives. A barrier to implementation was that alcohol screening was perceived as difficult. While the 15-method was perceived as effective in reducing the patients’ alcohol use and cost effective, participants expressed uncertainty about the long-term effects.

**Conclusions:**

The 15-method provides structure for treatment of alcohol use disorders and is described by general practitioners and heads as a promising approach. Being able to offer treatment for alcohol dependence may increase the uptake of alcohol interventions in primary care.

**KEY POINTS**
- Little attention has been given to develop treatment models for alcohol use disorders that are adapted to primary care settings.
- This study describes how an innovation, the 15-method, a stepped care model for treatment of alcohol use disorders in primary care was perceived.
- The 15-method provides structure for treatment of alcohol use disorders in primary care and is described by general practitioners and heads as a promising approach.
- Being able to offer treatment for alcohol dependence may increase the uptake of alcohol interventions in primary care.

## Introduction

Only a small minority of all individuals with alcohol use disorders (AUD) seek treatment. An important reason for this is the stigma associated with losing control over drinking and seeking treatment in specialist addiction clinics [1]. One way to reduce stigma and increase treatment seeking is to offer alcohol interventions in primary care. This is also in line with current discussions in the field of alcohol treatment [2,3]. Most research on alcohol interventions in primary care have up until now focused on secondary prevention – screening and brief interventions (SBI), which have been found efficacious for individuals with hazardous and harmful alcohol use [4]. There is however no evidence for the efficacy of SBI for individuals who have developed alcohol dependence [5]. However, little attention has been given to develop models that are adapted to primary care settings that also include treatment of alcohol dependence. Existing models have mainly focused on severe alcohol dependence [6–9]. In particular, there is a lack of research on treatment of individuals with moderately severe dependence.

For the purpose of offering interventions for the full spectrum of AUD in primary care, from hazardous alcohol use to alcohol dependence, we have developed the 15-method [10]. The 15-method starts with a brief intervention where after, if needed, the patient proceeds to interventions that are more extensive, including pharmacological and brief psychological treatment. The model consists of three steps: 1) identification of problem drinking and brief advice [4]; 2) assessment, with a 30 min feedback session [11,12]; 3) four sessions based on Cognitive Behavioural Therapy (CBT) and motivational interviewing [13,14]. Each session contains a theme with the aim to facilitate behaviour change: goal setting, self-monitoring of alcohol consumption, identifying risk situations and problem solving. These sessions can be combined with pharmacological treatment; acamprosate, disulfiram, nalmefene or naltrexone [15]. The name ‘the 15 method’, refers to that the length of sessions is 15 min, and that the final two steps of the intervention target patients who score above 15 points on the Alcohol Use Disorder Identification Test (AUDIT) [16]. Shared decision-making between the general practitioner (GP) and the patient guides the choice of type of treatment, treatment goal and intensity of treatment provided [17]. To offer treatment goals beyond the traditional focus on abstinence only is one important factor in broadening the focus for treatments for AUD, and also improving treatment seeking [1,18]. Moreover, reduction of alcohol use, rather than abstinence only, is associated with improved functioning, and improved physical and psychological health [19,20].

In previous publications we have reported the results from a six month and 12 month follow up of a randomized controlled trial (RCT), where we compared outcomes after treatment in primary care according to the 15-method to outpatient specialist addiction treatment [21,22]. In total, 288 patients fulfilled the ICD-10 criteria for alcohol dependence and were included in the trial. The interventions in primary care were delivered by the regular GPs, who received eight hours of training in the 15-method. The training consisted of lectures on the manual, combined with skills training including role-play of cases. In total, 29 GPs at 12 different primary care centers delivered the treatment. As most participants in the trial were recruited via advertisement, the treatment started on step 2, with the feedback session. In both groups of patients, a reduction in alcohol consumption, severity of dependence and drinking problems was seen at six and 12 months follow up compared to baseline, with no significant differences between the groups. Treatment in specialist care thus was not superior to treatment in primary care at either follow up, even if non-inferiority for primary care could not be concluded as the confidence interval exceeded the pre-specified non-inferiority limit of 50 grams of alcohol per week. We concluded that the 15-method is a promising approach for treatment of alcohol dependence in primary care.

Though large efforts have been made to increase the uptake, implementation of alcohol interventions in primary care has progressed slowly over the past 40 years [23]. Barriers to implementation that have been identified at the staff level are lack of knowledge and training [24–28], doubts about whether the interventions are effective [25,26], the opinion that pharmacological treatment for alcohol dependence is difficult to manage [26,27] and would not fit in the time frames of primary care [27]. Factors identified in facilitating the implementation are; training [25,29–31], financial incentives [31,32], on-the-job experience [31] and local champions [33]. The need for interventions tailored for the specific medical context of primary care has been emphasized [34].

In order to bridge the gap between interventions shown to be effective in clinical trials and the implementation of these interventions in regular clinical practice, the factors that can facilitate the implementation process need to be further investigated. This is especially true regarding factors influencing the staffs’ acceptance and usage of interventions. Diffusion of Innovation Theory, developed by Rogers [35], is one of the most used theories for studying how the utilization of innovations spreads. The theory seeks to explain factors influencing the rate of adoption and thus the external validity of new innovations. A key component is how potential adopters perceive the characteristics of an innovation. Diffusion of Innovation Theory was used as a theoretical framework in this study to explain the attributes of a promising innovation.

### Objectives

The overall objective of the present study was to explore how GPs and heads of clinics in primary care perceive the characteristics of an innovation, the 15-method, a stepped care model for treatment of AUD.

## Methods

### Trial design

A qualitative interview study.

### Research team

The research team consisted of: SWF – PhD and clinical psychologist, AH – Associate professor and registered psychotherapist, SA – Professor and MD, and MJ – Assistant professor and RN. SWF, AH and SA have extensive experience from alcohol research, working clinically with treatment of AUD and training clinical staff in treatment of AUD. MJ has extensive experience in qualitative analysis. The composition of the research team in relation to profession and research experience, enable reflexivity as well as minimizing the risk to draw premature conclusions based on a preunderstanding of the studied area.

### Participants

The participants were recruited among the GP’s and heads of the units that delivered the primary care treatment in the previously described RCT. The participants were chosen strategically from five units, which were geographically diverse in terms of north and south location, and also type of area; inner city, suburb or local suburban. In total, ten participants were approached of whom all agreed to participate. Three were both head of the unit and worked as a GP (Table 1).

**Table 1.** Table of participants.

Participant	Unit	Position	Gender	Follow up interview
1	A	Head / General practitioner	Female	Yes
2	A	General practitioner	Female	No
3	B	Head / General practitioner	Female	Yes
4	B	General practitioner	Female	No
5	C	Head	Female	Yes
6	C	General practitioner	Female	No
7	D	Head / General practitioner	Male	Yes
8	D	General practitioner	Male	Yes
9	E	Head	Female	No
10	E	General practitioner	Male	Yes

### Data collection

Data was collected via individual interviews that were conducted at two different time-points during the RCT. The first round of interviews (*n* = 10) were conducted during the autumn of 2013, after the participants had undergone training in the 15-method but before inclusion of the first patient. The second round of interviews (*n* = 6) were conducted six months later, during the spring 2014. Four participants could not be reached at the second round as they had changed positions and were no longer working at the unit. An open-ended, semi-structured interview guide was used, based on the theoretical framework of Diffusion of Innovation Theory, where the process of adoption is influenced by the following five factors:

1. Relative Advantage - The extent the innovation is seen as better than the idea or program it replaces.
2. Compatibility – How consistent the innovation is with values, experiences and needs of the adopters.
3. Complexity – How difficult the innovation is to understand and/or use.
4. Trialability – The extent to which the innovation can be tested before a commitment to adopt it.
5. Observability – The degree to which the innovation provides observable results [35].

The adoption factor ‘Trialability’ was not explored, as data was collected during a clinical trial and hence the participants did not have the choice whether to adopt the method or not. The interviews covered different aspects of the participants’ views on alcohol problems, interventions in primary care settings and the 15-method.

The interviews lasted 30–60 min and were conducted at the primary care units where the participants worked. All interviews were digitally recorded and transcribed verbatim. When reading the interview transcripts, the data from the interviews were considered sufficient to obtain information power [36].

### Data analysis

The analysis was conducted using theoretical thematic analysis as described by Braun and Clarke [37]. In order to get familiarized with the data, the transcripts were read repeatedly, and codes were created by the first and last author. Any discrepancies were solved through discussions, until consensus was reached. The codes were then grouped accordingly to coherence in topic relevant to the adoption factors in Diffusion of Innovation Theory, and themes were thereby constructed. The construction of the themes was discussed in the whole research team with few and easily solved discrepancies between the members.

### Ethics

The study was approved by the regional ethics board in Stockholm, 2012-11-07, ref: 2012/1760-31/1 and 2018/1355-2. All participants were given verbal and written information about the study and gave written consent.

## Results

The results are organized according to the theoretical framework, where two themes were identified for relative advantage, three for complexity and four themes for compatibility and observability respectively.

### Relative advantage compared to current clinical practice for AUD

#### Interventions for AUD meets a need among the patients in primary care

The majority of participants identified a need among existing patients in their clinics to receive treatment for AUD in primary care, especially for patients who do not want to seek or be referred to specialist addiction care. A majority of the participants expressed that this group can benefit from receiving treatment in primary care:

There are many who could consider going to a general practitioner but nothing else, and in that case coming to us, is what will work (Participant 10).

Several participants emphasized the need for uninterrupted patient flows in their clinics. They described that the patients’ reluctance to attend specialist addiction care is a barrier to work with alcohol interventions in primary care, leaving the GP:s with few options on how to manage these problems.

#### The 15-method gives a structure

In general, the participants were very positive to the manual of the 15-method and the use of the method. They mentioned a previous lack of structure and routines for alcohol interventions. A majority of the participants expressed that they had found it difficult to manage treatment for AUD due to a lack of local treatment programs and guidelines:

We have not had an actual local treatment program for less severe alcohol use disorders […] Well, you ask (the patient about their alcohol use), but it is a little bit troublesome because what to do when you get the answer? Do you start treatment with Antabuse? Well, then the patient comes here maybe once a week and receive these pills. This means a lot of pills and the patient might not be quite satisfied and you don’t have a really good treatment plan. That is what I feel is lacking (Participant 8).

The participants expressed how the 15-method filled this gap and provided a structure that contributed to increasing their self-esteem and enjoyment of working with this topic.

### Compatibility with existing values, experiences and needs of the adopters

#### A need for training in AUD interventions

A lack of knowledge was discussed as a previous barrier to working with AUD. The participating GPs were positive about the day of training and emphasized that they appreciated learning more about how to ask questions about alcohol use, using the questionnaires and the structure of the manual:

I think that I learned a great deal of things and got a lot of good pointers and reminders how I can ask these questions and follow these questionnaires. It is very stringent and well planned, so I think there is a red thread in it in a very positive way (Participant 7).

Especially the spirit of motivational interviewing appealed to many of the participants. Also, the need for more training in the techniques of behavior change were mentioned, especially how to raise the question of alcohol use initially, and how to engage patients in behavioral change and in treatment. Several expressed a need for more skills training on this topic:

The difficult part is to establish trust and get them onboard. And those who come now are already onboard. So that bit, in future, I would like to have more of (Participant 2).

Several participants made a point of the difference between applying the 15-method to treatment seeking patients as in the RCT, compared to a regular clinical practice where they meet patients who do not actively seek alcohol treatment.

#### More feasible to implement as a team

Most participants expressed that the 15-method would be more suitable to implement within a team of different professions, for example with nurses, rather than done by GPs only as the case in the RCT. Other professions were mentioned as better suited to deliver the psychological intervention:

We do not do any type of conversational life-style changing treatment in any area so therefore this feels like a quite big step. What we have is a life-style nurse with life-style interventions, so that would in a way be more natural (Participant 10).

Reasons given for this preference was, as in the quote above, that it was not seen as the role of GPs to deliver psychological interventions. Another reason given was that GPs are not financially reimbursed for delivering psychological interventions, which is the case for nurses. One further reason expressed was that the care for other diseases, as diabetes or asthma, is organized in teams.

#### Regional health care managers seen as important

Priorities made by the regional health care managers determine which type of services the primary care units get funded for. The participants expressed the need to prioritize requests from the managers in order to keep the budget:

There is a certain order of priorities if you follow what the health care managers wants, and what the managers want is what we get money for, and what we get money for we need to prioritize because otherwise it is not financially viable […] Now life-style factors are becoming more and more relevant (Participant 4).

There is an increased attention from the managers to focus on healthy lifestyle issues in the clinical work. Some participants expressed that working with alcohol interventions was a requirement from the managers, while others did not see it as a requirement but rather an area that the managers encouraged primary care to work more with.

#### Patient responsible to seek help

Several participants stated that they saw it as the patients’ responsibility to seek help for their excessive alcohol use. The participants expressed that the lack of time for GPs puts a higher responsibility on the patient to actively seek help:

We are pressured for time, and I believe many doctors put a little responsibility on the patient. I have asked this question, you maybe even mention there is help to be had. And if the patient says no, well then you let it go (Participant 3).

Several participants also emphasized that it was difficult as a GP to make time to engage patients in seeking treatment.

### Complexity

#### Easy to use

The participants found the 15-method easy to understand and use, especially after on-the-job experience of treating a couple of patients. All described how they found the 15-method to work well in the primary care setting. The method provided a structure and a help to engage with the patients.

I think it has worked well, because you now have a structure of how to meet these patients, and the more patients you meet, the more confident you get. And the patients have been positive to the program. In general, I would say it has actually worked well (Participant 8).

#### Timeframes

The timeframe of the visits evoked diverse responses - some said the visits fitted within 15 min, while others stated the visits were possible to deliver within the timeframes available in primary care, which is up to 30 min, but not within 15 min as suggested in the manual:

The first half an hour visit is never any problem. Then it depends on the patient, but that is the case irrespective of what it is (Participant 8).

Many participants expressed that one difficulty in regular clinical practice was to fit the revisits according to the suggested timeframe in the manual since the schedule often is fully booked a long time ahead

#### Alcohol screening seen as difficult

The participants stated they already ask patients they meet in their clinical work, outside the RCT, about their alcohol use. Often, they used targeted screening of patients with chronic diseases as hypertension, diabetes or psychiatric disorders. It was mentioned as important to make the question of alcohol use relevant to the patient in order to avoid violations of patient integrity:

The fact that you actually asked the question and tried to get the patient to realize that is was a relevant question. Why, if you come and have a high blood pressure, why on earth do you talk about alcohol (Participant 4)?

Some participants reported that they asked a larger number of patients about their alcohol use following participation in the study, especially female patients and also that they were more perceptive of the patients’ answers.

Several of the participants mentioned that the perceived difficulty in asking patients about their alcohol use was an important barrier to implementing the 15-method more broadly in clinical practice. One reason for this was lack of routines regarding alcohol screening:

There is no good structure, for example we do not offer these screening questionnaires in the waiting room, and we do not have any routines for how to catch it (patients’ alcohol use) so there is a lack of routines I would say (Participant 8).

In addition, a lack of time, lack of skills/knowledge about alcohol screening and also reluctance to ask as heavy alcohol use was seen as a stigmatized topic were mentioned as barriers to asking about the patients’ alcohol use.

### Observability

In the first round of interviews the participants were positive to the possible effects of the 15-method, but said it was difficult to evaluate before they had worked with it clinically. In the second round of interviews the participants stated more positive opinions about different aspects of the effects of the 15-method.

#### Effective in reducing alcohol use

The participants said the patients had reduced their alcohol use and were satisfied with the treatment. A majority of the participants stated the 15-method had an effect on the patients’ alcohol use:

I see that they drink less, if they are being honest and aren’t lying to me, so I think many of us have said that it actually seems like they drink less (Participant 1).

This participant expressed some uncertainty whether the patients were honest, but also states that colleagues have said that their patients had reduced their alcohol consumption.

#### Cost effective

Moreover, the 15-method was described as cost-effective, referring both to the aspect that the session time is brief and that the interventions are inexpensive. This participant also emphasized that the 15-method is cost-effective on a societal level:

Yes, I believe it is cost effective. On all levels, even on a societal level (Participant 7).

#### Alcohol diary

Of the different components of the manual, the alcohol diary was mentioned as a particularly valuable tool by several of the participants. One of the participants mentioned that her patients said the diary was helpful:

I believe both my two (patients) have said that the alcohol diary was good. Then you are a bit picky and ask them when they get back, yes but this day, how come it was four glasses on Wednesday (Participant 1)?

The alcohol diary also seems to work as a tool for the GP to ask more specific questions about the patients’ alcohol use.

#### Long term effects uncertain

The 15-method was seen as effective, however many stated uncertainty whether the effects would be maintained over a longer period of time:

Yes, I actually think so, I do. At least in the short-term, whether there is a more long-term effect I do not know (Participant 1).

Several participants mentioned the outcome data from the RCT as important in order to assess the effects, and to guide the decision whether to implement the 15-method in regular clinical practice.

## Discussion

The aim of the present study was to explore how GPs and heads of clinics perceive the characteristics of an innovation, the 15-method a stepped care model for treatment of AUD in primary care. Data collection and analyzes were guided by the factors identified to influence the adoption of an innovation according to the Diffusion of Innovation Theory: Relative Advantage; Compatibility; Complexity and Observability [35]. Data was collected via interviews with GPs and heads of primary care units who delivered treatment in a RCT [21,22]. The aim of the RCT was to compare treatment of alcohol dependence in primary care according to a manual we have developed, the 15-method, to treatment in a specialized addiction unit.

Overall, we found high levels of satisfaction with the 15-method and its application among the GPs and heads. The participants described a previous lack of routines in how to work with patients with alcohol dependence, where the training and manual provided them with the needed knowledge and structure. Moreover, a need for offering this type of treatment in primary care was described in order to meet a need among patients they already meet. The participants also expressed the view that the 15-method was effective in reducing the patients’ alcohol use, confirming the findings from the RCT [21,22]. In line with previous studies in the field [25,29], the participants described it as more feasible to implement the 15-method in regular clinical practice as a team with different professions rather than conducted by GPs only, as was the case in the RCT. The approach with the regular clinical staff delivering the interventions differs from the Screening, Brief Intervention, and Referral to Treatment (SBIRT) grant program in the US, where specialist staff was hired to provide the interventions in primary care [38,39]. This also emphasizes the need to tailor implementation efforts to suit different health care systems.

The results from this study are encouraging for efforts to implement treatment for alcohol use disorders in primary care, which has been the goal for several decades for organisations as the World Health Organisation (WHO) and many national health ministries. As previously mentioned, the challenge for many years has been the uptake of SBI in routine practice. One important factor for this reluctance among GPs is a perceived lack of expertise in managing AUD [23]. Given that screening will identify not only hazardous consumers, but also those who have developed alcohol dependence, practitioners may hesitate to raise the initial question of alcohol use in the absence of routines to handle dependence and the known difficulties of referring patients to specialist treatment [3,40]. The results from this study and the RCT are therefore important - GPs with a brief training in a stepped care manual can achieve similar treatment outcomes as addiction specialists for the large group of socially well-adjusted individuals with alcohol dependence. Importantly, the clinicians were positive to the 15-method and described it as easy to use in the context of primary care.

The staff in this study raised concerns about asking patients about their alcohol use, if they do not specifically seek treatment for alcohol problems. A lack of routines, training and skills regarding how to address alcohol consumption were expressed as important barriers, confirming previous studies [41,42]. SBI is part of the 15-method but was not included in the RCT. The findings highlight the need to focus both on offering skills training in SBI for primary care staff, and also to develop new tools to support clinicians in asking about alcohol. For example, digital interventions can be one such new tool [43]. Clinicians need updated information on the role of alcohol for a large spectrum of common ailments that bring patients to their health centres. This information is also important in making questions about alcohol consumption understandable to the patients. Some participants also raised concerns about screening, reasoning that it is the patients’ responsibility to seek treatment for AUD. Again, the fact that alcohol causes a large health burden whether the patients are alcohol dependent or not needs to be emphasized [44]. However, for the dependent patients the comment highlights the importance of also developing new treatment approaches that are feasible to implement in primary care, and that individuals with AUD find acceptable to seek and engage in.

For future implementation efforts it is also important to take into account organizational factors. This far, many efforts have focused on individual clinicians’ skills and attitudes [25,29]. The results from this study highlight the importance of regional health care policy, requests from management and also the importance of financial incentives to support behavioral change among clinicians.

## Limitations

One limitation is that data was collected during a RCT, and thus does not necessarily reflect regular clinical practice. However, the aim of the study was to explore how the characteristics of an innovation, the 15-method is perceived by the staff. The participants were all from primary care units in areas with medium to higher socio-economic status, and we lack views of units in areas with lower socio-economic status. Descriptive data as age or level of experience of the participants were unfortunately not systematically collected. Moreover, four participants could not be reached for the follow up interview as they had left their practices, which poses a risk of attrition bias. The study design was to conduct the first interviews after training, but before inclusion of any patients in the RCT and the follow-up interviews six months later with the same participants. Therefore, it was not possible to recruit new participants to replace the ones that we were unable to reach. However, among those who completed both interviews, all units, roles; GP, head respectively GP/head and both genders were represented, which indicates the results reflect diverse views, and thus are valid.

## Conclusion

The 15-method provides a needed structure for treatment of alcohol use disorders in primary care and is a promising approach. Moreover, the implementation of interventions for hazardous use may well be benefited by offering treatment for alcohol dependence in primary care. Future implementation efforts should focus more on the role of regional health care policy.
